# Healthcare use in commercially insured youth with mental health disorders

**DOI:** 10.1186/s12913-022-08353-z

**Published:** 2022-07-26

**Authors:** Julie Hugunin, Maryann Davis, Celine Larkin, Jonggyu Baek, Brian Skehan, Kate L. Lapane

**Affiliations:** 1 Clinical and Population Health Research PhD Program, Morningside Graduate School of Biomedical Sciences, University of Massachusetts Chan Medical School, Worcester, USA; 2 Department of Population and Quantitative Health Sciences, Albert Sherman Center, University of Massachusetts Chan Medical School, 368 Plantation Street, MB 01605 Worcester, USA; 3 Department of Psychiatry, University of Massachusetts Chan Medical School, Worcester, USA; 4 Department of Emergency Medicine, University of Massachusetts Chan Medical School, Worcester, USA; 5 Department of Pediatrics, University of Massachusetts Chan Medical School, Worcester, USA

**Keywords:** Mental health services, Primary care, Transition to adulthood

## Abstract

**Background:**

The objective of this study is to describe age-related patterns of outpatient healthcare utilization in youth and young adults with mental health disorders.

**Method:**

We used the IBM^®^ MarketScan^®^ Commercial Database to identify 359,413 youth and young adults (12–27 years) with a mental health disorder continuously enrolled in private health insurance in 2018. Exploratory analysis was used to describe patterns of outpatient healthcare use (e.g., primary, reproductive, mental health care) and therapeutic management (e.g., medication prescriptions, psychotherapy) by age. Period prevalence and median number of visits are reported. Additional analysis explored utilization patterns by mental health disorder.

**Results:**

The prevalence of outpatient mental health care and primary care decreased with age, with a larger drop in primary care utilization. While 74.0-78.4% of those aged 12–17 years used both outpatient mental health care and primary care, 53.1–59.7% of those aged 18–27 years did. Most 18–19-year-olds had a visit with an internal medicine or family medicine specialist, a minority had a pediatrician visit. The prevalence of medication management increased with age, while the prevalence of psychotherapy decreased.

**Conclusions:**

Taken together, this descriptive study illustrates age-related differences in outpatient healthcare utilization among those with mental health disorders. Additionally, those with the most severe mental health disorders seem to be least connected to outpatient care. This knowledge can inform efforts to improve utilization of healthcare across the transition to adulthood.

**Supplementary Information:**

The online version contains supplementary material available at 10.1186/s12913-022-08353-z.

## Background

The onset of mental health disorders is before the age of 25 years in 75% of those diagnosed during their lifetime, making late adolescence and young adulthood a critical time for early detection and intervention [1]. Thus, utilization of appropriate health services throughout adolescence and into early adulthood is likely essential for improved outcomes in this population. However, evidence indicates that young adults have the lowest rates of healthcare utilization among all age groups, including mental [2, 3] and preventative [4] health service use. In 2019, almost half of young adults in the United States with mental health disorders received no mental health services [5]. Untreated, mental health disorders can result in houselessness, involvement with the justice system, incarceration, vulnerability to crime, and high risk of suicide [6, 7].

A growing body of research indicates that barriers in the transition from pediatric to adult care may result in utilization drop-offs among young adults [8]. Due to a fragmented mental healthcare system, youth with mental health disorders typically interact with multiple systems and agencies, making it particularly difficult to navigate the transition to adult care [9, 10]. They must quickly learn, often independently, how to navigate changing insurance eligibility, vocational and housing opportunities, and eligibility for additional services [10]. Additionally, youth and young adults with mental health disorders can experience significant delays in psychosocial development that can compromise family relationships, quality of friendships, and success in school/workplace, [10] and can impact one’s ability for self-care and participation in routine health care decision-making [11].

Clinical guidelines [11] and key federal reports [7–9] recognize that youth with mental or behavioral health conditions experience substantial adversity during the shift from pediatric to adult health care. The American Psychiatric Association emphasizes that these “transition-age youth” are underserved in current mental health systems [12]. Yet most research addressing the transition from pediatric to adult care has not focused on populations with mental illness and utilization of mental health services across this age range [13]. The few studies that have examined age-related patterns of care in youth and young adults with mental health disorders are limited in scope. Decreased mental health treatment rates at 18–19 years as compared to 16–17 years, and rising rates at 20–25 years have been described [14]. However, this cross-sectional study uses data over two decades old and is limited to youth utilizing specialty mental health treatment programs [14]. Another study reports decreased use of mental health services beginning in mid-adolescence, however this study does not account for mental health need, including the presence of a mental health disorder [15]. A more recent study of youth (7–19 years) with bipolar disorder reports a general decline in healthcare use, including individual psychotherapy and home-based care [16].

To our knowledge, no studies have explored age-related changes in primary care utilization in youth and young adults with mental health disorders. Given the historic inequitable treatment of physical health conditions in those with mental health disorders [17, 18] and the mortality disparity experienced by this population, [19] a better understanding of primary care utilization patterns in this population is essential to improving health outcomes.

To address this research gap, we aimed to describe age-related healthcare utilization patterns in youth and young adults with mental health disorders. We used a national data resource of healthcare claims on commercially insured youth and young adults to describe outpatient primary care and mental healthcare utilization patterns, medication management, reproductive health visits and substance use treatment. This was a descriptive research study, as such no specific hypotheses were tested.

## Methods

This study was deemed non-human subjects research by the Institutional Review Board of the University of Massachusetts Chan Medical School.

### Stakeholder engagement

To ensure that the research generated relevant knowledge and data interpretation involved real-world insight, we engaged stakeholders from across the country. These stakeholders included representatives from the Young Adult Advisory Board from the Transitions to Adulthood Research Center at UMass Chan Medical School, The National Federation of Families, Got Transition^®^ (from the National Alliance to Advance Adolescent Health), Mental Health America, Commonwealth Medicine, and Reliant Medical Group. Two virtual stakeholder forums were held. In the first meeting, we solicited feedback on our planned research goals and approach. We modified key decisions based on their input (e.g., range of ages included, selection of mental health disorders to include). During the second forum, we shared preliminary results and solicited feedback on how to interpret our findings and what additional analyses may be useful to stakeholders.

### Dataset

This study used the most recent data available at the time of the study initiation from the IBM^®^ MarketScan^®^ Commercial Database (2018) [20]. This administrative claims database is the largest collection of de-identified, patient-level data in the United States. It includes information related to health service use across the continuum of care, including inpatient, outpatient, and medication prescriptions. Those included in this database are privately insured employees and their spouses/partners and dependents.

### Study sample

The study sample included youth and young adults, defined as those 12–27 years, with a mental health disorder (i.e., major depressive disorder, bipolar disorder, schizophrenia, other psychotic disorders, anxiety disorders/phobias, post-traumatic stress disorder, and disruptive disorders including conduct and intermittent explosive disorder). We explored this sample based on stakeholder input. Patients with at least 2 outpatient claims or at least 1 inpatient claim with ICD-10 codes (in any diagnosis field in any claim in 2018) indicating a mental health disorder were identified (Table S1). An individual may have had co-morbid conditions and thus could be classified as having more than one mental health disorder.

Exclusion criteria included those: (1) without continuous insurance enrollment throughout 2018 (*n* = 118,017), (2) whose mental health/substance abuse claims were not covered/not present in the data resource (*n* = 30,621), (3) without drug coverage (*n* = 19,764), and (4) missing health plan type (*n* = 6,755). These exclusion criteria were applied so that period prevalence of mental health service use could be explored. The final sample included 359,413 youth and young adults with a mental health disorder continuously enrolled in private health insurance in 2018 (Figure S1).

### Health service measures

The key exploratory measure was outpatient health service use. Outpatient healthcare utilization included mental health care, primary care, reproductive health visits, and substance use care in those with co-morbid substance use disorder. Any outpatient healthcare utilization was explored. We also created a 4-level categorial measure of care utilization: mental health and primary care, mental health care only, primary care only, and none. Psychiatric residential facilities (as defined in the database) were also explored.

Outpatient mental healthcare was defined using provider type (e.g., psychiatry, child psychiatry, psychiatric nurse), place of service (e.g., office, outpatient, community mental health center), and mental health service outpatient sub-category codes (Table S2). Current Procedural Terminology (CPT) codes and place of service codes were used to specify type of outpatient utilization as evaluation/management/diagnosis, psychotherapy, psychiatric partial hospitalization (intensive day programs), or other.

Primary care utilization was defined using provider type (e.g., internal medicine, family practice, pediatrician, nurse practitioner), place of service (e.g., office, outpatient, federally qualified health center), and Current Procedural Terminology (CPT) codes (Table S2). For claims with a primary care physician, primary care was further characterized as pediatric, adult, or family by provider type (pediatrician, internal medicine, and family practice, respectively). An outpatient reproductive health visit was defined using provider type (obstetrics, gynecology, midwife), place of service (e.g., office, outpatient, federally qualified health center), and Current Procedural Terminology (CPT) codes (Table S2). Use was explored only in females.

Among those with co-morbid substance use, substance use treatment was explored. Co-morbid substance use was identified using at least 2 outpatient claims or at least 1 inpatient claim with ICD-10 codes (in any diagnosis field) indicating substance use disorder (Table S3). Substance use treatment included care received in a substance abuse facility and medication management for substance use disorder via generic names of medications (Table S3).

### Additional measures

Medication use was determined using outpatient drug claims grouped by the IBM Micromedex^®^ RED BOOK^™^ therapeutic class indicating antidepressant, anxiolytics, mood stabilizers, and antipsychotics [21]. Prescriptions for any benzodiazepine and any tricyclic antidepressant were also explored. These were chosen given that benzodiazepines should be used cautiously due to the possibility of developing dependence and tricyclic antidepressants can lead to severe side effects, [22] and while these drugs may be indicated in some cases, a large prevalence of these prescriptions in adolescents would be concerning [22]. A binary indicator for any drug claim for each therapeutic class, any benzodiazepine, and any tricyclic antidepressant prescribed in 2018 was created. Management of mental health disorder was categorized as psychotherapy and medication management, psychotherapy only, medication management only, or none.

Additional measures include age, sex, type of health insurance, and medical complexity. Age was categorized into eight two-year strata (12–13, 14–15, 16–17, 18–19, 20–21, 22–23, 24–25, and 26–27 years) based on age at insurance enrollment in 2018. Sex available in the database was limited to male and female. Healthcare plan type was categorized as high deductible health plan (HDHP)/consumer driven health plans (CDHP), basic/ major medical/ comprehensive plan, preferred provider organization, and all others (exclusive provider organization, health maintenance organization, point-of-service). Medical complexity was measured by the Pediatric Medical Complexity Algorithm (less conservative version 3.1), a validated algorithm used to classify youth as complex chronic (> 1 body system involved or ≥ 1 condition is progressive or ≥ 1 condition is malignant), non-complex chronic (1 body system involved and the condition is not progressive or malignant), and without chronic disease (no body system indicators present) [23]. This publicly available algorithm was developed by the Center of Excellence on Quality of Care Measures for Children with Complex Needs. We removed the mental health disorders used to define our sample from the algorithm.

### Data analysis

Descriptive analysis was used to explore covariates, health care utilization patterns, and therapeutic management of mental health disorder by age group. Period prevalence and the median number of outpatient healthcare visits per year were calculated for outpatient service use by type of outpatient service use. We estimate the proportion of outpatient healthcare by provider specialty (pediatrician, family medicine physician, internal medicine physician) by age group. The behavioral health management (i.e., psychotherapy, filled prescriptions) of mental health disorder was evaluated by age. Because of the large sample size, even trivial differences would be statistically significant. For this reason, instead of using p-values, we considered absolute differences of 5% as clinically relevant. Additional analysis explored patterns of utilization by mental health diagnosis. Data was analyzed using SAS® software, Version 9.4.

## Results

### Sample characteristics

The average age was 19.9 years, 64.3% were female, 63.6% were not medically complex (aside from the mental health disorder), and 51.5% had a preferred provider health care plan (Table 1). Anxiety disorders (74.0%) were the most common clinician-assessed psychiatric diagnosis, followed by major depressive disorder (57.8%). Schizophrenia (1.3%) and other psychotic disorders (1.4%) were the least common. More than one mental health disorder was observed in 46.9%. Clinician-assessed co-morbid substance use was observed in 6.4%. Sample characteristics differed by age. The prevalence of anxiety disorders, bipolar disorder, and co-morbid substance use was higher in the older age groups relative to the younger age groups, whereas the prevalence of disruptive disorders was lower.

**Table 1:** Individual-level characteristics of youth and young adults with mental health disorders, by age group (2018)

	**Age group, years**										
	12–13 (*n* = 14,417)	14–15 (*n* = 37,861)	16–17 (*n* = 64,838)	18–19 (*n* = 52,838)	20–21 (*n* = 54,134)	22–23 (*n* = 54,574)	24–25 (*n* = 49,218)	26–27 (*n* = 31,533)		Overall (*n* = 359,413)	
**Mental health disorder**^**a**^											
Schizophrenia	0.2	0.3	0.5	1.1	1.8	2.1	2.1	1.2		1.3	
Other psychotic disorders	0.8	1.0	1.1	1.6	1.8	1.6	1.5	0.7		1.4	
Bipolar disorder	2.6	3.7	5.2	8.3	10.2	10.7	10.5	8.3		8.0	
Post-traumatic stress disorder	4.0	4.4	5.0	5.4	6.3	6.8	6.5	6.1		5.7	
Major depressive disorder	42.9	54.5	62.4	61.7	60.0	57.7	55.4	53.2		57.8	
Anxiety disorders	72.3	72.3	71.7	74.0	74.4	74.8	75.1	77.5		74.0	
Disruptive disorders	14.7	10.7	6.4	2.3	1.1	0.9	0.7	0.5		3.6	
**Co-morbid substance use disorder**	1.7	3.1	5.3	6.1	7.7	8.6	8.8	5.0		6.4	
**Female**	53.4	61.5	63.6	65.0	65.1	64.7	65.6	68.6		64.3	
**Plan type**											
Basic/ major medical/comprehensive	2.4	2.6	2.8	2.8	2.9	2.9	3.4	3.3		2.9	
Preferred provider organization	51.6	50.9	50.8	51.5	51.7	52.3	52.1	51.1		51.5	
High-deductible/consumer-driven	22.9	23.3	23.7	23.7	23.4	22.2	21.6	19.6		22.8	
All other health plans	23.1	23.2	22.7	22.0	21.9	22.3	22.9	26.0		22.8	
**Pediatric medical complexity**											
Non-chronic	67.3	66.6	67.5	65.8	64.2	62.0	59.0	55.8		63.6	
Non-complex chronic	22.7	23.1	22.2	22.3	22.5	23.0	23.9	24.9		23.0	
Complex chronic	10.1	10.3	10.3	11.8	13.3	15.0	17.2	19.3		13.4	

^a^Not mutually exclusive, thus will add up to more than 100%; anxiety disorders include generalized anxiety disorder, separation anxiety disorder, specific or social phobia; disruptive disorders include conduct disorders and intermittent explosive disorder

### Outpatient health care utilization patterns

The majority of individuals had some outpatient mental health care use (91.3%), two-thirds had some primary care use (68.3%), and many females had a reproductive health visit (21.8%) (Table 2). The majority of outpatient mental health care use was related to psychotherapy and evaluation, management, or diagnosis, and the minority was partial psychiatric hospitalization or another reason; outpatient psychotherapy is described in behavioral health management below. Any outpatient mental health care and any primary care use was less common in the older age groups relative to the younger age groups. However, the prevalence of reproductive health visits in females was higher in older age groups (14–15 years: 7.8%, 16–17 years: 16.6%, 18–19 years: 23.1%, 26–27 years: 30.0%).

**Table 2:** Past year healthcare utilization rates in those with mental health disorders, by age group (2018)

	Age group, years								
	12–13 (*n* = 14,417)	14–15 (*n* = 37,861)	16–17 (*n* = 64,838)	18–19 (*n* = 52,838)	20–21 (*n* = 54,134)	22–23 (*n* = 54,574)	24–25 (*n* = 49,218)	26–27 (*n* = 31,533)	Overall (*n* = 359,413)
**Outpatient mental health care**									
% with any mental health care	96.0	95.7	94.7	91.8	90.2	89.1	87.9	86.8	91.3
% with any related to evaluation/management/diagnosis	83.2	84.3	83.8	83.0	82.7	81.0	79.4	78.1	82.0
% with partial psychiatric hospitalization	1.6	1.8	1.5	1.1	1.2	1.2	1.1	0.6	1.2
% with other mental health visit	0.7	0.6	0.6	0.5	0.6	0.6	0.5	0.4	0.6
**Primary care**									
% with any	81.7	80.3	78.3	65.8	62.5	61.2	62.1	63.5	68.3
**Reproductive health visit in females**^**a**^									
% with any	2.7	7.8	16.6	23.1	24.5	26.5	27.8	30.0	21.8
**Psychiatric residential treatment center**									
% with any	0.7	0.8	0.8	0.5	0.5	0.5	0.4	0.3	0.6
**Substance use care for those dually diagnosed**									
% with any	90.4	79.4	68.9	62.4	63.1	68.2	70.7	67.2	67.8

^a^Operational definition in Table S2; only includes an outpatient visit with an obstetrics and gynecology physician or midwife (primary care physicians and additional specialties providing reproductive care are not included)

**Fig. 1:**
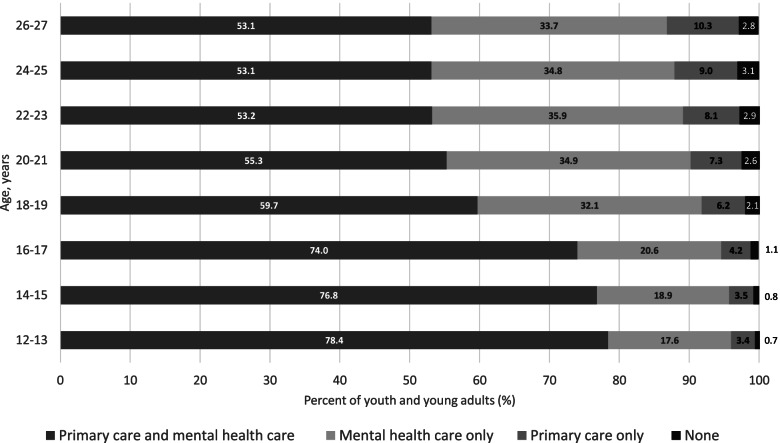
Outpatient healthcare use in those with mental health disorders, by age group (2018)

As shown in Fig. 1, the prevalence of outpatient mental health care only and primary care use only was higher in the older age groups, and patients in the older age groups had lower prevalence of using both primary care and outpatient mental health care. While 74.0-78.4% of those aged 12–17 years utilized both outpatient mental health care and primary care, 53.1–59.7% of those aged 18–27 years did. Overall, use of outpatient mental health care only was seen in 29.6% and primary care only was seen in 6.6%, while using both primary care and outpatient mental health care use was seen in 61.7% of individuals.

**Fig. 2:**
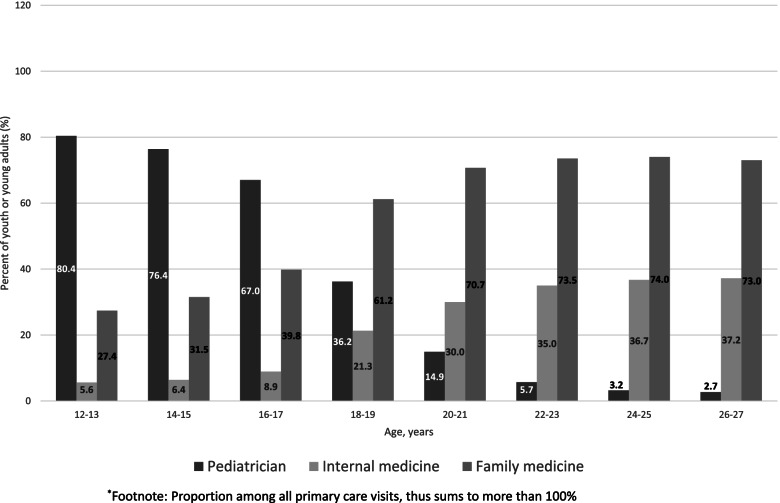
Proportion of primary care delivered by physician specialty in those with mental health disorders, by age group (2018)

Of those with a primary care visit, 89.2% had at least one visit with a pediatrician, internal medicine, or family medicine physician. Among patients aged 12–13 years, 80.4% had a visit with a pediatrician, 27.4% with a family medicine physician, and 5.6% with an internal medicine physician (Fig. 2). Among patients aged 16–17 years, fewer had pediatric visits and more had adult visits. Among those aged 26–27 years, 2.7% had a visit with a pediatrician, 73.0% with a family medicine physician, and 37.2% with an internal medicine physician.

### Behavioral health management

Any prescription filled for an antidepressant, anxiolytic, mood stabilizer, or antipsychotic was seen in 72.9%, while only 59.6% had a psychotherapy visit (Table S7). Overall, 65.0% filled a prescription for an antidepressant, 24.0% for an anxiolytic, 17.1% a mood stabilizer, and 12.9% an antipsychotic prescription. Among those less than 18 years, 5.9% filled a prescription for a benzodiazepine and 2.3% for a tricyclic antidepressant. Prescription fills for each type of psychiatric medication were more common in older age groups, except for antipsychotic and tricyclic antidepressant prescriptions, which remained relatively consistent across age groups. The median number of psychotherapy visits per year was 7 (Table S6).

**Fig. 3:**
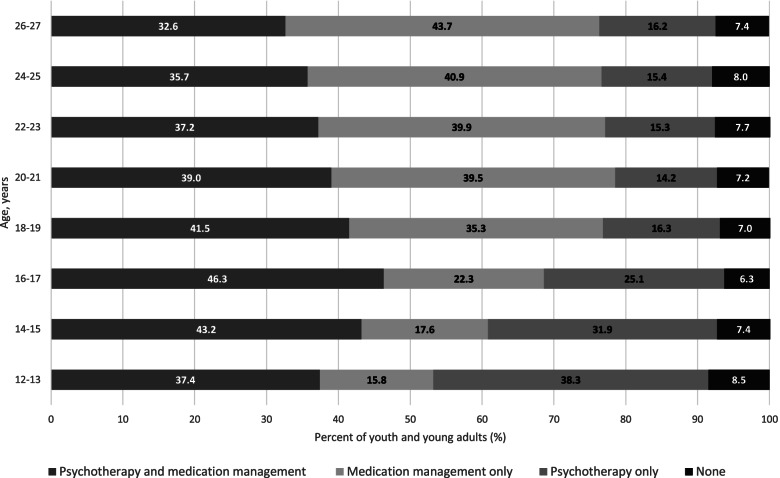
Behavioral health management of those with mental health disorders, by age group (2018)

Psychotherapy was more common among younger age groups, while medication management was more common in older age groups (Fig. 3). Medication management without psychotherapy was 15.5% among those aged 12–13 years, 35.5% among those aged 18–19 years, and 43.7% among those aged 26–27 years (Table S7).

Of the 6.4% of the sample with clinician-assessed co-morbid substance use (Table 1), 67.8% received substance use care (Table 2), and 16.5% filled prescriptions to medically manage the substance use disorder (Table S7). Among those with co-morbid substance use disorder, older age groups had a higher prevalence of prescriptions filled for substance use disorder (16–17 years: 1.8%, 18–19 years: 8.3%, 20–21 years: 15.9%, 22–23 years: 23.5%, 24–25 years: 29.7%).

### Additional analysis by mental health disorder

Differences in healthcare utilization patterns were observed by mental health disorder (Table S5). Notably, the prevalence of primary care use was lowest in those with schizophrenia (52.3%) and other psychotic disorders (56.9%). Additionally, benzodiazepine prescriptions in those less than 18 years were highest in those with schizophrenia (18.6%), other psychotic disorder (14.6%), and bipolar disorder (11.7%). The prevalence of co-morbid substance use was highest in those with other psychotic disorders (24.4%), schizophrenia (19.9%), and bipolar disorder (15.5%). These were also the disorders with the lowest prevalence of substance use care (schizophrenia: 59.7%, bipolar disorder: 62.8%, other psychotic disorders: 63.2%). Medication prescriptions for substance use disorder were seen in 9.8% of those with schizophrenia, 7.4% of those with other psychotic disorders, and 16.8% of those with bipolar disorder.

## Discussion

This exploratory study documents a notable drop in the prevalence of outpatient healthcare use among those with mental health disorders at 18 years, especially in primary care use, as compared to those aged 12 to 17 years. Among those aged 18–19 years (and in older age groups), most had a visit with an internal medicine or family medicine specialist. The prevalence of psychotherapy was lower in young adults as compared to younger age groups, while medication management was higher. These data indicate important age-related differences in healthcare use, which can be used to focus interventions to improve access to care as youth transition from pediatric to adult care.

Previous literature has described insurance changes as a potential reason for declining rates of outpatient healthcare use among emerging adults [10]. Yet, in this population of privately insured youth and young adults, outpatient healthcare utilization was significantly less common among the older age groups, indicating that insurance alone is not sufficient to ensure healthcare use. Our stakeholder groups pointed to differing eligibility requirements and system changes at 18 years. These changes may be beyond insurance policy and instead be related to institutional norms or system processes [10]. Evidence indicates that in countries with universal healthcare, about 75% do not access adult mental health services upon reaching the upper age limit of child mental health services [24]. Early transitional planning would likely be beneficial, as would flexibility in allowing a youth to remain in a pediatric practice past 18 years of age. Additionally, a systematic review of international literature focused on improving the transition from pediatric to adult care highlights patient education, joint pediatric/adult clinics, and specific young adult clinics as potential successful interventions [25]. Other policy reforms may include a formal process for tracking and managing the transition and formal handovers in care [26].

Our data suggest that the transition from pediatric to adult care may be happening as early as 14–15 years in some youth with mental health disorders. While our data did not provide explanations for this finding, stakeholders suspect that for some youth, visiting clinics created for children may feel uncomfortable. It may also feel difficult to discuss certain topics when a parent is present at the visit, even if they are not in the room. For other youth with mental health disorders, it may be a daunting task to create a relationship with a new provider, and they may prefer to remain with their pediatrician for as long as possible, or they may drop out of primary care [27]. The current healthcare system does not account for these factors, potentially leading to declines in care during this transition point. A report from the American Academy of Pediatrics, the American Academy of Family Physicians, and the American College of Physicians emphasizes the Six Core Elements [28] as a structural process to guide providers in the healthcare transition [11]. Adolescents and young adults continue to report that these key transitional elements are not being met [29]. Additionally, this process currently lacks guidelines for behavioral health clinicians [30] and it is unknown if it meets the needs of those with mental health disorders [31]. Creative models of transitional care are needed for this population, focusing on flexibility and meeting the patient where they are at.

 A successful transition to primary care may help to provide social support as youth emerge into adulthood. On the frontline of early detection and intervention, primary care providers can provide a gateway to specialty services, act as a “medical home” and an advocate for potentially vulnerable patients, offer continuity of care, promotion of healthy habits, prevention of chronic diseases, and are associated with decreased acute healthcare service utilization [32–35]. Young adults with lived experience, key members of our stakeholder advisory board, described commitments to school, work, and moving for college as major barriers to utilizing primary care and psychotherapy. Fewer patients in the young adult age groups had health care claims for psychotherapy as compared to younger age groups. A key sentiment was that they are often in ‘survival mode,’ trying to meet their commitments while managing the symptoms of a mental health disorder. Parents are often less involved in their care and the youth may experience more autonomy. With this may come the need to navigate the healthcare system on their own, a task which can be exceedingly difficult for a young adult with a mental health disorder. Earlier intervention, education, and connection to community supports could help to address risk factors which may emerge during young adulthood, often concurrently when many youths are lost in the transition to adult care.

Youth and young adults with specific mental health disorders such as schizophrenia, bipolar disorder, and other psychotic disorders may require specialized intervention, and seemed to experience exceptional levels of undertreatment in the current analyses. Primary care utilization was lowest among those with these disorders. Additionally, while co-morbid substance use was highest in this group, they also had the lowest rates of substance use care. Experts have called for such conditions to be designated as a health disparity, given the inequities in care, stigma, and discrimination documented in this population [17, 19, 36]. Wraparound services and Coordinated Specialty Care are models of care especially beneficial to this population [37, 38]. Yet private health insurers typically do not provide this level of care, though many youth in their system require it. Additionally, primary care and transitional planning remain under-utilized and under-emphasized in these models, though data indicate their benefits [34, 35]. Additional research among transition-age youth with serious mental illnesses such as schizophrenia, bipolar disorders, and other psychotic disorders is needed.

Our study has several limitations to consider. The sample captures youth and young adults continuously enrolled in private insurance in 2018 and who have utilized healthcare while enrolled. Thus, this study is not representative of all youth and young adults with mental health disorders. For instance, it is possible that those with mental health disorders are less likely to have continuous insurance enrollment and thus would not be included in this study. Indeed, this sample includes healthcare users and the data comes from administrative claims of those with private health insurance, thus the prevalence of services are overstated relative to the general population. For instance, the rate of outpatient mental healthcare utilization reported in this study (91.3%) is much higher than the rate reported by the U.S. Department of Health and Human Services in 2018 (37.3%) [39].

Additionally, we are limited to data within the IBM^®^ MarketScan^®^ Commercial Database. This database lacks racial/ethnic information, income information, educational attainment, county-level data, and other social determinants of health. Each of these factors may influence the age at which a mental health disorder is identified, as well as utilization patterns. This database also lacks information related to insurance changes. Given that young adults may move off their parent’s insurance during this time, it is possible that decreased outpatient utilization observed might be related to recent insurance changes in the older age groups. Additionally, we are limited to health care utilization represented within the claims database. For some, this may not include carve-out behavioral health care, out-of-pocket care, or care received in other settings such as schools, which may be essential sources of care. Thus, we do not have a complete understanding of utilization patterns.

The impact of the COVID-19 pandemic on youth mental health must also be considered when interpreting these findings. Disruptions to school and social supports have led to a mental health crisis in youth and young adults [40]. Additionally, preventative care has decreased because of delayed care during the pandemic [41]. Taken together, patterns of healthcare use in this population have likely changed.

This is a cross-sectional, observational study, thus no conclusions about causality can be made, and some age-related patterns may be cohort effects rather than period effects. However, this exploratory research fills a surprising gap, that of healthcare utilization patterns as youth and young adults with mental health disorders transition into adulthood. Given that in 2018 private health insurance coverage included 68.9% of adults aged 18–64 years, 54.7% of children aged 0–17 years, [42] and about 63% of young adults (18–25 years) with mental health disorders, [43] analyses from the IBM^®^ MarketScan^®^ database is representative of healthcare utilization for many youth and young adults with mental health conditions in the US.

## Conclusions

By focusing on those with mental health disorders, this study builds upon previous literature describing low healthcare utilization in young adults and challenges in the transition from pediatric to adult care. Continuous engagement in primary care and mental health care during the transition to adulthood is vital to improving healthcare quality and outcomes in youth and young adults with mental health disorders, yet this exploratory study describes age-related declines in outpatient care. Ultimately, in the context of existing knowledge, this study adds to literature emphasizing the need for creative approaches and increased investment in our youth and in mental health care. Now, more than ever, a collective effort is needed to address gaps in care for those with mental health disorders.

## Supplementary Information

**Additional file 1.**

## Data Availability

The data that support the findings of this study are available from IBM® Watson Health™ but restrictions apply to the availability of these data, which were used under license for the current study, and so are not publicly available. Data are however available from the authors upon reasonable request and with permission of IBM® Watson Health™.
