# Supplementary material for: Healthcare use in commercially insured youth with mental health disorders
Source: BMC Health Serv Res. 2022 Jul 26;22:952. doi: 10.1186/s12913-022-08353-z (PMC9323879; doi:10.1186/s12913-022-08353-z)
Supplement: Supplementary file 1 — Additional file 1. [file 12913_2022_8353_MOESM1_ESM.docx]

Healthcare use in commercially insured youth with mental health disorders

*Authors: Julie Hugunin, MD/PhD candidate; Maryann Davis, PhD; Celine Larkin, PhD; Jonggyu Baek, PhD; Brian Skehan, MD/PhD; Kate L. Lapane, PhD*

Supplemental Material Table of Contents

[Supplemental Tables – Methods 1](#_Toc107341286)

[Table S1. Sample definition 1](#_Toc107341287)

[Table S2. Outpatient health service utilization codes 2](#_Toc107341288)

[Table S3. Substance use codes 4](#_Toc107341289)

[Supplemental Figures 6](#_Toc107341290)

[Figure S1. Sample selection 6](#_Toc107341291)

[Supplemental Tables – Additional Analysis 7](#_Toc107341292)

[Table S5. Additional analysis by mental health disorder, 2018 7](#_Toc107341293)

[Table S6. Median number of visits per person per year by health care utilization type, 2018 10](#_Toc107341294)

[Table S7. Behavioral health management by age group, 2018 11](#_Toc107341295)

[References 12](#_Toc107341296)

# Supplemental Tables – Methods

| Table S1. Sample definition |  |
| --- | --- |
| **Mental health disorder** | **ICD-10 Code** |
| Schizophrenia | F20*, F25* |
| Other psychotic disorders | F23, F28, F29 |
| Bipolar disorder | F30*, F31* |
| Depressive disorders | F32*, F33*, F34.1 |
| Anxiety disorders and phobias | F40*, F41*, F93.0 |
| Post-traumatic stress disorder | F43.1 |
| Disruptive disorders | F91*, F63.81 |
| ^*^Applied to the to the 15 diagnosis codes on the Inpatient Admissions Table and to the 4 diagnosis codes on the Outpatient Services Table | |

| Table S2. Outpatient health service utilization codes | |
| --- | --- |
| **Description** | **Codes Used** |
| Outpatient Mental Healthcare Claim | **Any outpatient mental healthcare**  *Provider Type Codes (stdprov):*  21 (Mental health facilities) or 365 (Psychiatry) or 458 (Child psychiatry) or 824 (Psychiatric nurse) or 853 (Supportive therapist) or 860 (Psychologist)  *And Place of service codes (stdplac):*  11 (Office) or 12 (Patient Home) or 19 (Outpatient Hospital, off campus) or 22 (Outpatient Hospital, on campus) or 50 (Federally Qualified Health Center) or 95 (Outpatient, Not Elsewhere Classified)  *Or Service Sub-Category Code (svscat):*  30524 (Mental Health Physician OP Preventive Visits) or 30525 (Mental Health Physician OP Office Visits) or 30624 (Mental Health Professional OP Preventive Visits) or 30625 (Mental Health Professional OP Office Visits) or 30630 (Mental Health Professional OP Diagnostic Services) or 30518 (Mental Health Physician OP Behavioral Health Therapy) or 30618 (Mental Health Professional OP Behavioral Health Therapy)  *Or Place of service codes (stdplac):*  52 (Psych Facility Partial Hospitalization) or 53 (Community Mental Health Center)  **Evaluation/ management/ diagnosis**  *Provider Type Codes (stdprov): as above*  *And Place of service codes (stdplac):*  11 (Office) or 12 (Patient Home) or 19 (Outpatient Hospital, off campus) or 22 (Outpatient Hospital, on campus) or 50 (Federally Qualified Health Center) or 95 (Outpatient, Not Elsewhere Classified) or 53 (Community Mental Health Center)  *And CPT Codes (proc1)*^1,2^*:*  99201-99205 or 99211-99215 or 99241-99245 or 90791-90792  *Or Service Sub-Category Code (svscat):*  30524 (Mental Health Physician OP Preventive Visits) or 30525 (Mental Health Physician OP Office Visits) or 30624 (Mental Health Professional OP Preventive Visits) or 30625 (Mental Health Professional OP Office Visits) or 30630 (Mental Health Professional OP Diagnostic Services)  **Any psychotherapy**  *Provider Type Codes (stdprov): as above*  *And Place of service codes (stdplac):*  11 (Office) or 12 (Patient Home) or 19 (Outpatient Hospital, off campus) or 22 (Outpatient Hospital, on campus) or 50 (Federally Qualified Health Center) or 95 (Outpatient, Not Elsewhere Classified) or 53 (Community Mental Health Center)  *And CPT Codes (proc1)*^1,2^*:*  90832-90834 or 90836-90840 or 90846 or 90847 or 90849 or 90853 or 90887  *Or Service Sub-Category Code (svscat):*  30518 (Mental Health Physician OP Behavioral Health Therapy) or 30618 (Mental Health Professional OP Behavioral Health Therapy)  **Any psych facility partial hospitalization:**  *Place of service codes (stdplac):*  52 (Psych Facility Partial Hospitalization)  **Other:**  *Provider Type Codes (stdprov): as above*  *And Place of service codes (stdplac):*  11 (Office) or 12 (Patient Home) or 19 (Outpatient Hospital, off campus) or 22 (Outpatient Hospital, on campus) or 50 (Federally Qualified Health Center) or 95 (Outpatient, Not Elsewhere Classified)  *And CPT Codes (proc1)*^1,2^*:*  90845 or 90865 or 90867 or 90868 or 90869 or 90870 or 90875 or 90876 or 90880 or 90882 or 90885 or 90889 or 90899 or 90901 or 90911  *Or Place of service codes (stdplac):*  53 (Community Mental Health Center, not included above) |
| Outpatient Primary Care Claim | *Provider Type Codes (stdprov):*  204 (Internal Medicine) or 240 (Family Practice) or 400 (Pediatrician) or 822 (Nursing Services) or 825 (Nurse Practitioner) or 845 (Physician assistant)  *And Place of service codes (stdplac):*  11 (Office) or 12 (Patient Home) or 19 (Outpatient Hospital, off campus) or 22 (Outpatient Hospital, on campus) or 50 (Federally Qualified Health Center) or 95 (Outpatient, Not Elsewhere Classified)  *And CPT Codes (proc1)*^3^*:*  99201-99205 or 99211-99215 or 99218-99220 or 99224-99226 or 99241-99245 or 99304-99345 or 99347-99350 or 99354-99357 or 99374-99384 or 99391-99394 or 99401-99412 or 99420 or 99429 or 99455-99456 or 99432 or 99460 or 99461 |
| Reproductive Health Care Claim | *Provider Type Codes (stdprov):*  320 (Obstetrics and Gynecology) or 820 (Midwife)  *And Place of service codes (stdplac):*  11 (Office) or 12 (Patient Home) or 19 (Outpatient Hospital, off campus) or 22 (Outpatient Hospital, on campus) or 50 (Federally Qualified Health Center) or 95 (Outpatient, Not Elsewhere Classified)  *And CPT Codes (proc1)*^4^*:*  57170 or 58300 or 59400 or 59410 or 59425 or 59426 or 59430 or 59510 or 59515 or 59610 or 59614 or 59618 or 59622 or 77055–77057 or 77061-77063 or 77065-77067 or 87110 or 87270 or 87320 or 87490–87492 or 87620–87622 or 87624-87625 or 87810 or 88141–88143 or 88147 or 88148 or 88150 or 88152– 88154 or 88164–88167 or 88174 or 88175 or 99201-99205 or 99211-99215 or 99241-99245 or 99500-99501 |
| **Applied to IBM® MarketScan® Commercial Database, Outpatient Services Table** | |

| Table S3. Substance use codes | |
| --- | --- |
| **Description** | **Codes Used** |
| Substance use disorder  *Applied to the to the 15 diagnosis codes on the Inpatient Admissions Table and to the 4 diagnosis codes on the Outpatient Services Table* | *ICD-10 codes*^5–7^*:*  F10.10, F10.12*, F10.14-F10.92*, F10.94-F10.99, F11.10, F11.12*, F11.14-F11.99, F12.10, F12.12*, F12.15*-F12.22*, F12.25*-F12.92*, F12.95*-F12.99, F13.10, F13.12*, F13.14-F13.20, F13.22*-F13.99, F14.10, F14.12*, F14.14-F14.99, F15.10, F15.12*, F15.14-F15.20, F15.22*-F15.99, F16.10, F16.12*, F16.14-F16.99, F18.10, F18.12*-F18.99, F19.10, F19.12*, F19.14-F19.99, F55.0-F55.8, G62.1, I42.6, K29.2*, K70*, O35.4XX*-O35.5XX*, O99.31*-O99.32*, T40.0X1A, T40.0X3A, T40.0X4A, T40.0X5A, T40.1X1A, T40.1X3A, T40.1X4A, T40.2X1A, T40.2X3A, T40.2X4A, T40.2X5A, T40.3X1A, T40.3X3A, T40.3X4A, T40.3X5A, T40.4X1A, T40.4X3A, T40.4X4A, T40.4X5A, T40.5X1A, T40.5X3A, T40.5X4A, T40.5X5A, T40.601A, T40.603A, T40.604A, T40.605A, T40.691A, T40.693A, T40.694A, T40.695A, T407X1A, T407X3A, T407X4A, T407X5A, T408X1A, T408X3A, T408X4A, T40901A, T40903A, T40904A, T40905A, T40991A, T40993A, T40994A, T40995A, T43601A, T43603A, T43604A, T43605A, T43621A, T43623A, T43624A, T43625A, T43631A, T43633A, T43634A, T43635A, T43691A, T43693A, T43694A, T43695A, Z726 |
| Substance use care *Applied to IBM® MarketScan® Commercial Database, Outpatient Services Table* | *Provider Type Codes (stdprov):*  20 (Mental health/ chemical dep, Not Elsewhere Classified) or 22 (Chemical depend treatment ctr) or 23 (Mental health/ chem dep day care)  *Or Place of Service Codes (stdplac):*  55 (Residential Subst Abuse Facil) or 57 (Non-Resident Subst Abuse Facil)  *Or CPT Codes (proc1)*^8^*:*  *99408-99409*  *Or Service Sub-Category Code (svscat):*  31110-31769 (Substance abuse facility, physician, or professional)  Excluding*:* 31120, 31220, 31320, 31420, 31520, 31620 (Substance use ER) |
| Substance use medication management | *Generic names (gennme):*  Acamprosate  Disulfiram  Nalmefene  Naltrexone  Methadone  Buprenorphine |

|  |
| --- |
|  |

# Supplemental Figures

## Figure S1. Sample selection


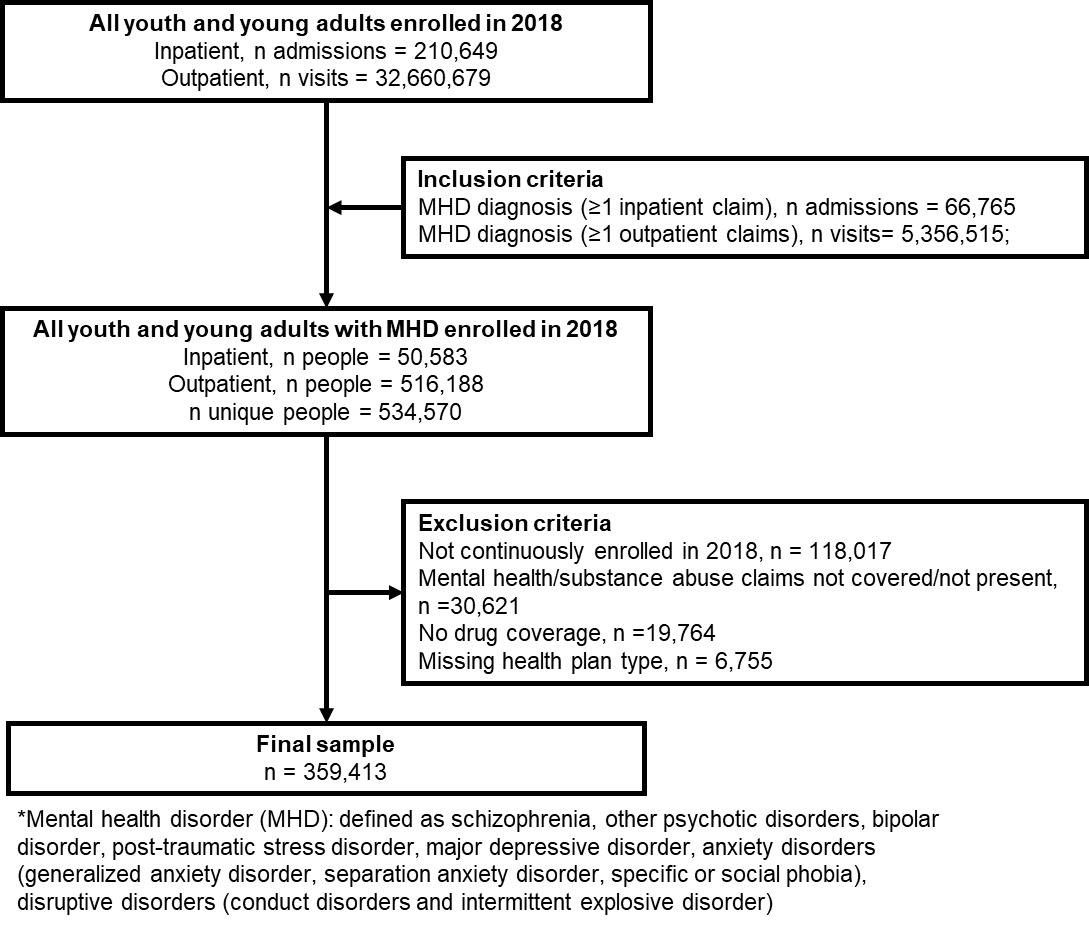


# Supplemental Tables – Additional Analysis

## Table S5. Additional analysis by mental health disorder, 2018

|  | | Mental Health Disorder^*^ | | | | | | | | | | | | | |
| --- | --- | --- | --- | --- | --- | --- | --- | --- | --- | --- | --- | --- | --- | --- | --- |
|  | | Schizophrenia  (n=4,601) | | Other psychotic disorders  (n=4,846) | | Bipolar disorder  (n=28,688) | | Post-traumatic stress disorder  (n=20,638) | | Major depressive disorder  (n=207,839) | | Anxiety disorders  (n=265,835) | | Disruptive disorders  (n=13,023) | |
| *Individual Characteristics* | |  | |  | |  | |  | |  | |  | |  | |
| **Co-morbid substance use disorder** | | 19.9 | | 24.4 | | 15.5 | | 11.7 | | 7.5 | | 5.7 | | 10.3 | |
| **Female** | | 34.7 | | 38.5 | | 62.2 | | 77.9 | | 66.1 | | 66.9 | | 32.4 | |
| **Plan type** | |  | |  | |  | |  | |  | |  | |  | |
| Basic/ major medical/ comprehensive | | 3.6 | | 3.1 | | 3.3 | | 3.3 | | 3.0 | | 2.8 | | 3.7 | |
| Preferred provider organization | | 51.3 | | 50.0 | | 52.7 | | 52.6 | | 51.2 | | 51.5 | | 53.4 | |
| High-deductible/consumer-driven | | 20.9 | | 20.7 | | 21.7 | | 21.5 | | 22.8 | | 22.9 | | 20.8 | |
| All other health plans | | 24.3 | | 26.2 | | 22.3 | | 22.6 | | 23.0 | | 22.9 | | 22.1 | |
| **Pediatric medical complexity** | |  | |  | |  | |  | |  | |  | |  | |
| Non-chronic | | 54.9 | | 57.7 | | 58.1 | | 59.2 | | 63.6 | | 62.7 | | 64.2 | |
| Non-complex chronic | | 25.5 | | 24.0 | | 24.3 | | 24.1 | | 22.9 | | 23.5 | | 23.2 | |
| Complex chronic | | 19.6 | | 18.3 | | 17.6 | | 16.7 | | 13.5 | | 13.8 | | 12.6 | |
| *Past year healthcare utilization rates* | |  | |  | |  | |  | |  | |  | |  | |
| **Outpatient healthcare utilization** | |  | |  | |  | |  | |  | |  | |  | |
| Primary care and any mental health care | | 48.8 | | 52.1 | | 60.8 | | 66.5 | | 62.9 | | 63.3 | | 68.9 | |
| Mental health care only | | 42.1 | | 36.4 | | 33.1 | | 29.4 | | 30.0 | | 28.2 | | 26.3 | |
| Primary care only | | 3.4 | | 4.8 | | 3.7 | | 2.6 | | 5.2 | | 6.8 | | 3.0 | |
| None | | 5.7 | | 6.7 | | 2.4 | | 1.5 | | 1.9 | | 1.8 | | 1.9 | |
| **Outpatient mental health care** | |  | |  | |  | |  | |  | |  | |  | |
| % with any mental health care | | 90.9 | | 88.5 | | 93.9 | | 95.9 | | 92.8 | | 91.4 | | 95.1 | |
| % with any related to  evaluation/management/diagnosis | | 87.3 | | 84.4 | | 90.4 | | 85.6 | | 85.2 | | 83.2 | | 86.6 | |
| % with partial psychiatric hospitalization | | 3.5 | | 4.1 | | 2.9 | | 2.8 | | 1.7 | | 1.1 | | 2.2 | |
| % with other mental health visit | | 3.1 | | 1.9 | | 1.3 | | 1.0 | | 0.7 | | 0.5 | | 1.0 | |
| **Primary care** | |  | |  | |  | |  | |  | |  | |  | |
| % with any | | 52.3 | | 56.9 | | 64.5 | | 69.0 | | 68.1 | | 70.1 | | 71.8 | |
| **Reproductive health care in females** | |  | |  | |  | |  | |  | |  | |  | |
| % with any | | 20.0 | | 21.5 | | 27.6 | | 25.0 | | 21.9 | | 21.9 | | 15.0 | |
| **Psychiatric residential treatment center** | |  | |  | |  | |  | |  | |  | |  | |
| % with any | | 1.2 | | 1.3 | | 1.0 | | 0.9 | | 0.7 | | 0.5 | | 1.3 | |
| **Substance use care for those dually diagnosed** | |  | |  | |  | |  | |  | |  | |  | |
| % with any | | 59.7 | | 63.2 | | 62.8 | | 65.9 | | 67.1 | | 66.9 | | 69.8 | |
| *Behavioral health management* | |  | |  | |  | |  | |  | |  | |  | |
| **Behavioral health management** | |  | |  | |  | |  | |  | |  | |  | |
| Any medication management | | 89.4 | | 85.0 | | 90.5 | | 74.8 | | 78.0 | | 75.4 | | 61.5 | |
| Any psychotherapy | | 63.3 | | 61.5 | | 66.4 | | 78.9 | | 63.6 | | 58.4 | | 72.8 | |
| Psychotherapy and medication management | | 58.7 | | 55.2 | | 60.9 | | 57.4 | | 47.2 | | 40.7 | | 44.2 | |
| Medication management, no psychotherapy | | 30.7 | | 29.8 | | 29.6 | | 17.4 | | 30.8 | | 34.6 | | 17.4 | |
| Psychotherapy, no medication management | | 4.6 | | 6.3 | | 5.5 | | 21.5 | | 16.4 | | 17.7 | | 28.6 | |
| None | | 6.0 | | 8.7 | | 4.0 | | 3.7 | | 5.6 | | 7.0 | | 9.9 | |
| **Medication management** | |  | |  | |  | |  | |  | |  | |  | |
| % filled prescription for antidepressant | | 56.5 | | 57.3 | | 63.3 | | 66.0 | | 73.3 | | 68.0 | | 47.0 | |
| % filled prescription for antianxiety | | 32.4 | | 32.0 | | 36.1 | | 33.5 | | 24.5 | | 27.2 | | 15.3 | |
| % filled prescription for mood stabilizer | | 43.9 | | 35.1 | | 63.4 | | 28.8 | | 17.5 | | 16.4 | | 21.2 | |
| % filled prescription for antipsychotic | | 81.7 | | 67.3 | | 55.4 | | 23.7 | | 13.7 | | 10.8 | | 28.7 | |
| % filled prescription for benzodiazepine  (in those <18 years) | | 18.6 | | 14.6 | | 11.7 | | 7.6 | | 5.8 | | 6.8 | | 5.0 | |
| % filled prescription for a tricyclic  (in those <18 years) | | 3.0 | | 2.6 | | 2.4 | | 2.9 | | 2.3 | | 2.5 | | 1.6 | |
| **Substance use care for those dually diagnosed** | |  | |  | |  | |  | |  | |  | |  | |
| % with any medication management | | 9.8 | | 7.4 | | 16.8 | | 18.0 | | 15.8 | | 17.3 | | 3.4 | |
| ^*^An individual might be categorized as having more than one condition and thus would be included in more than one category | | | | | | | | | | | | | | | |

| Table S6. Median number of visits per person per year by health care utilization type, 2018 | | |
| --- | --- | --- |
|  | Median # visits per person per year | 25^th^ and 75^th^ percentile |
| **Outpatient mental health care** |  |  |
| Any mental health care per year | 5 | 3, 13 |
| Evaluation/management/diagnosis per year | 3 | 1, 5 |
| Psychotherapy per year | 7 | 3, 15 |
| Partial psychiatric hospitalization | 5 | 2, 11 |
| Other mental health care | 6 | 1, 19 |
| **Primary care** | 2 | 1, 4 |
| **Reproductive health care in females** | 1 | 1, 2 |

| Table S7. Behavioral health management by age group, 2018 | | | | | | | | |  |
| --- | --- | --- | --- | --- | --- | --- | --- | --- | --- |
|  | Age group, years | | | | | | | |  |
|  | 12-13 | 14-15 | 16-17 | 18-19 | 20-21 | 22-23 | 24-25 | 26-27 | Overall |
| **Behavioral health management** |  |  |  |  |  |  |  |  |  |
| Any medication management | 53.2 | 60.8 | 68.6 | 76.8 | 78.6 | 77.1 | 76.6 | 76.4 | 72.9 |
| Any psychotherapy | 75.7 | 75.1 | 71.4 | 57.7 | 53.3 | 52.5 | 51.2 | 48.8 | 59.6 |
| Psychotherapy and medication management | 37.4 | 43.2 | 46.3 | 41.5 | 39.0 | 37.2 | 35.7 | 32.6 | 39.8 |
| Medication management only | 15.8 | 17.6 | 22.3 | 35.3 | 39.5 | 39.9 | 40.9 | 43.7 | 33.1 |
| Psychotherapy only | 38.3 | 31.9 | 25.1 | 16.3 | 14.2 | 15.3 | 15.4 | 16.2 | 19.8 |
| None | 8.5 | 7.4 | 6.3 | 7.0 | 7.2 | 7.7 | 8.0 | 7.4 | 7.3 |
| **Medication management** |  |  |  |  |  |  |  |  |  |
| % filled prescription for antidepressant | 47.4 | 55.2 | 63.0 | 69.6 | 70.2 | 67.5 | 66.0 | 66.1 | 65.0 |
| % filled prescription for antianxiety | 10.1 | 13.5 | 16.9 | 22.9 | 27.4 | 29.5 | 31.4 | 32.5 | 24.0 |
| % filled prescription for mood stabilizer | 7.8 | 9.9 | 12.7 | 16.7 | 19.4 | 21.5 | 22.1 | 20.0 | 17.1 |
| % filled prescription for antipsychotic | 10.9 | 11.8 | 12.4 | 13.6 | 14.5 | 13.9 | 13.6 | 9.5 | 12.9 |
| % filled prescription for benzodiazepine | 3.6 | 4.8 | 7.0 | 12.3 | 16.9 | 20.7 | 23.5 | 25.9 | 14.9 |
| % filled prescription for tricyclic antidepressant | 1.5 | 2.1 | 2.5 | 2.8 | 3.2 | 3.3 | 3.6 | 3.3 | 2.9 |
| **Substance use care for those dually diagnosed** |  |  |  |  |  |  |  |  |  |
| % with any medication management | 0.0 | 0.9 | 1.8 | 8.3 | 15.9 | 23.5 | 29.7 | 24.3 | 16.5 |

# References

1. Shain B, Barron-Seabrook S, Chang J, Wang K, Berland DI, O’Keefe D. *CPT Code Training Module*.; 2018. https://www.aacap.org/App_Themes/AACAP/docs/clinical_practice_center/business_of_practice/cpt/2018_CPT_module_revised_March_2018.pdf

2. American Psychiatric Association. *CPT Primer for Psychiatrists*.; 2018. https://www.psychiatry.org/File Library/Psychiatrists/Practice/Practice-Management/Coding-Reimbursement-Medicare-Medicaid/Coding-Reimbursement/cpt-primer-for-psychiatrists.pdf

3. Mangione-Smith R. Continuity of Primary Care Quality Measure. Kaiser Permanente. Accessed May 6, 2021. https://www.kpwashingtonresearch.org/our-research/our-scientists/rita-mangione-smith-md-mph/measurement-tools-research-dr-rita-mangione-smith

4. HEDIS Coding Document, MVP Health Care. 2018 Coding Reference Guide for OBGYN. Published online 2018. Accessed October 5, 2021. http://www.ncqa.org/hedis-quality-measurement/hedis-measures/hedis-2018

5. Heslin KC, Elixhauser A, Steiner CA. Hospitalizations Involving Mental and Substance Use Disorders Among Adults, 2012. *Healthc Cost Util Proj Stat Briefs*. Published online 2015. Accessed August 13, 2021. https://www.ncbi.nlm.nih.gov/books/NBK310986/

6. Heslin KC, Elixhauser A. Mental and Substance Use Disorders Among Hospitalized Teenagers, 2012. *Natl Inpatient Sample*. Published online 2016:2012. Accessed August 13, 2021. http://www.samhsa.gov/data/sites/default/files/2013MHDetTabs/NSDUH-

7. Owens PL, Fingar KR, McDermott KW, Muhuri P, Heslin KC. *Inpatient Stays Involving Mental and Substance Use Disorders, 2016* .; 2019. Accessed August 13, 2021. https://www.hcup-us.ahrq.gov/reports/statbriefs/sb249-Mental-Substance-Use-Disorder-Hospital-Stays-2016.jsp

8. SAMHSA. Coding for Screening and Brief Intervention Reimbursement. Published 2020. Accessed October 5, 2021. https://www.samhsa.gov/sbirt/coding-reimbursement

9. Voss E, Ma Q, Ryan P. The impact of standardizing the definition of visits on the consistency of multi-database observational health research. *BMC Med Res Methodol*. 2015;15(1). doi:10.1186/S12874-015-0001-6
